# Prevalence of Mood and Anxiety Disorders Among Adults Seeking Care in Primary Healthcare Centers in Cordoba, Argentina.

**DOI:** 10.3389/fpsyt.2020.00232

**Published:** 2020-03-27

**Authors:** María Soledad Burrone, Rubén Alvarado, Lisandro D. Colantonio, Julio E. Enders, Roberto Ariel Abeldaño Zuñiga, Eliecer Valencia, Ezra Susser, Ruth A. Fernández

**Affiliations:** ^1^Instituto de Ciencias de la Salud, Universidad de O´Higgins, Rancagua, Chile; ^2^Escuela de Salud Pública, Facultad de Ciencias Médicas de la Universidad Nacional de Córdoba, Córdoba, Argentina; ^3^Faculty of Medicine, University of Chile, Santiago, Chile; ^4^Department of Epidemiology, University of Alabama at Birmingham, Birmingham, AL, United States; ^5^División de Estudios de Posgrado, Universidad de la Sierra Sur – Consejo Nacional de Ciencia y Tecnología, Oaxaca, México; ^6^Department of Epidemiology, Columbia University, New York, NY, United States; ^7^Division of Health Policy and Behavioural Science, New York State Psychiatric Institute, New York, NY, United States

**Keywords:** mental disorders, prevalence, Argentina, consultant, primary care

## Abstract

**Objective:**

To estimate the prevalence of mood and anxiety disorders among adults seeking care in primary healthcare centers in Cordoba city, Argentina.

**Methods:**

Cross-sectional analysis of a random sample of adults 18–69 years of age seeking care for general health problems in public (i.e., government-funded) primary healthcare centers in Cordoba city, Argentina in 2010–2011. Mood and anxiety disorders were assessed in the participants’ lifetime, and in the last 12 months and 30 days using the World Mental Health Composite International Diagnostic Interview 3.0, and defined following the International Classification of Diseases, tenth revision.

**Results:**

Overall, 1,067 participants were included in the current analysis [mean age 35.6 (SD 13.2) years, 83.7% female]. The lifetime, 12-month and 30-day prevalence of any mood or anxiety disorder was 40.4% [95% confidence interval (95%CI) 37.4–43.4%], 20.1% (17.8–22.7%) and 7.8% (6.2–9.6%), respectively. The prevalence of anxiety disorders was higher compared to mood disorders when assessed in the participants’ lifetime [29.7% (95%CI 27.0–32.5%) versus 19.3% (17.0–21.8%)], and in the last 12 months [14.9% (12.8–17.2%) versus 8.7% (7.1–10.6%)] and 30 days [5.8% (4.5–7.4%) versus 2.3% (1.5–3.4%)]. Age and marital status-adjusted odds ratios for any mood or anxiety disorder in the participants’ lifetime and in the last 12 months and 30 days comparing women versus men were 1.19 (95%CI 0.85–1.67), 1.70 (1.07–2.69), and 2.26 (1.02–5.00), respectively.

**Conclusion:**

The prevalence of mood and anxiety disorders is high among adults seeking care in primary healthcare centers in Cordoba city, particularly among women. Integration of primary and mental health services is warranted.

## Introduction

Mental health is a fundamental and inseparable component of health and is directly related to the individual, family, and community well-being (1–3). The burden of mental disorders has become increasingly relevant as this causes a high degree of individual and social suffering (3). It has been estimated that 15.4% of adults had a mood or anxiety disorder in the last 12 months (4). It has been also estimated that mental disorders, including anxiety and mood disorders, accounts for about 6% of all disability-adjusted life-years lost worldwide, representing more than all neurological and substance use disorders combined (5).

The primary health care strategy involves the organization of health systems in three levels of care with progressive complexity (i.e., the primary, secondary, and tertiary levels) (6–9). The aim of the primary health care strategy is maximizing the health in a community through an efficient use of local resources and following equity and solidarity principles (6–9). By maximizing the overall level of health in the community, the primary health care strategy can contribute to promote the economic and social development of low- and middle-income countries (7, 8). Healthcare services utilization is high among individuals with mental disorders (10, 11). Also, prior studies suggest that the prevalence of mental disorders may be high among people seeking care for general health problems in the first level of care (i.e., primary healthcare centers) (12–15). Data on the prevalence of mental disorders in adults seeking care in primary healthcare centers were used to support the inclusion of mental health services into the primary health care strategy in Chile, a country in which this integration was successfully accomplished (16–19).

The main objective of the current analysis was to estimate the prevalence of mood and anxiety disorders among adults seeking care in public (i.e., government-funded) primary healthcare centers in Cordoba city, the second largest city in Argentina. Public healthcare centers provide free services to all residents in Argentina (i.e., universal coverage), including unemployed, immigrants, and those with lower income and resources. If the prevalence of mood and anxiety disorders is high in adults seeking care in public primary healthcare centers in Cordoba city, this would support the need for a local integration of mental health services into the primary health care strategy. Integrating mental health services into the primary health care strategy could contribute to reduce the burden of mental disorders by increasing the diagnosis, referral, and treatment among individuals with mental disorders while promoting an efficient use of resources (20, 21).

## Methods

### Study Design

We conducted a cross-sectional analysis of adults 18 to 69 years of age seeking care for general health problems in public primary healthcare centers in Cordoba city, Argentina in 2010–2011. Participants were randomly selected using a probabilistic, stratified, multi-stage sampling design. Strata were defined to represent geographic areas of Cordoba city which have different socioeconomic characteristics. Within each stratum, primary healthcare centers, including community health centers and primary healthcare units, were randomly selected with a probability proportional to their total number of patients 18 to 69 years of age treated. At least one community health center and one primary healthcare unit were selected within each stratum. The final number of primary healthcare centers selected from each stratum was defined to be proportional to the total number of centers across strata. In each primary healthcare center, a fixed proportion of all patients 18 to 69 years of age with an appointment in a single day were randomly selected from the patient list and invited to participate in the current study. The process was repeated on different days until at least 50 participants were enrolled. Overall, 133 individuals who were invited to participate from the current study refused or did not complete the data collection (see below). These individuals were replaced by another patient randomly selected from the patient list. The target population size for the current study was 1,000 participants. The study protocol was approved by the Hospital Nacional de Clinicas Institutional Review Board, Córdoba, Argentina and all participants provided written informed consent.

### Data Collection

Trained interviewers administered a brief survey to collect sociodemographic data (age, sex, marital status, being married or in a marriage-like relationship, and nationality) and the computer-assisted World Mental Health Composite International Diagnostic Interview (CIDI) 3.0 (22). The CIDI 3.0 is a fully-structured interview designed to be administered by trained lay interviewers. Training of interviewers is required to ensure compliance with a number of field quality assurance principles and scoring rules. The CIDI 3.0 includes a screening module and a diagnosis module, which is composed of sections assessing mood, anxiety, substance use, childhood, and other disorders in the lifetime, last 12 months and last 30 days. The computer-assisted version of the CIDI facilitates the selection of sections to be included in the analysis. The CIDI allows to classify disorders following the International Classification of Diseases, tenth revision (ICD-10) or the Diagnostic and Statistical Manual of Mental Disorders, fourth edition. Prior studies have shown that the CIDI has acceptable to good concordance with clinician diagnoses of mental disorders, although the concordance varies across mental disorders groups (23–25). In an analysis of 143 World Mental Health Survey Initiative participants ≥18 years of age from Spain, Italy, France and the US, the area under the curve for 12-month prevalence comparing the CIDI versus clinician diagnoses was 0.88 for anxiety disorders and 0.83 for mood disorders (22).

For the current analysis, interviewers were trained by a mental health practitioner certified as an official trainer for the computer-assisted CIDI 3.0. Only CIDI sections for diagnosis of mood and anxiety disorders were administered to participants (in addition to the screening module) as these are the most common mental disorders in adults (5). Mood and anxiety disorders were classified according to the ICD-10. Mood disorders included mania (ICD-10 codes F30.xx excluding F30.0), hypomania (ICD-10 code F30.0), severe depressive episode (ICD-10 codes F32.2 and F32.3), moderate depressive episode (ICD-10 code F32.1), mild depressive episode (ICD-10 code F32.0), and dysthymia (ICD-10 code F34.1). Anxiety disorders included agoraphobia without panic disorder (ICD-10 code F40.02), social phobia (ICD-10 codes F40.1x), panic disorder (ICD-10 codes F40.01 and F41.0), generalized anxiety disorder (ICD-10 code F41.1), obsessive compulsive disorder (ICD-10 codes F42.x), and post-traumatic stress disorder (ICD-10 codes F43.1x).

### Statistical Analysis

Sociodemographic characteristics of the study population were summarized using mean and standard deviation for continuous variables and proportion for binary/categorical variables, overall and among men and women, separately. We calculated the prevalence and 95% confidence intervals (CI) of any mood or anxiety disorder in the participants’ lifetime (i.e., the lifetime prevalence) and in the last 12 months (i.e., the 12-month prevalence) and 30 days (i.e., the 30-day prevalence). The lifetime, 12-month and 30-day prevalence were also calculated for mood and anxiety disorders, separately, for each specific disorder analyzed (e.g., agoraphobia without panic disorder), and for having both a mood and an anxiety disorder (i.e., comorbidity). In addition to the analysis in the overall population, the calculation of the lifetime, 12-month and 30-day prevalence was repeated stratified by age, sex, and being married or in a marriage-like relationship status. Analyses were not repeated stratified by nationality as the vast majority of participants were from Argentina. The lifetime, 12-month and 30-day prevalence of mood and anxiety disorders across subgroups defined by age, sex, and being married or in a marriage-like relationship status were compared using the Fisher’s exact test given the small number of observations in some cells.

Logistic regression models were used to estimate odds ratios and 95% CIs for any mood or anxiety disorder, and for mood and anxiety disorders, separately, in the participants’ lifetime and in the last 12 months and 30 days associated with age, sex, and being married or in a marriage-like relationship status. Models included adjustment for age, sex, and being married or in a marriage-like relationship status, simultaneously. All analyses were conducted using STATA 11.2 (StataCorp LP, College Station, TX). Statistical significance was defined as a two-sided alpha level <0.05.

## Results

### Characteristics of the Study Population

Overall, 1,067 participants were included in the current study, of whom 893 (83.7%) were women (**Table 1**). The mean (standard deviation) age was 35.6 (13.2) years in the overall study population, 35.2 (13.1) years among women, and 37.9 (13.9) years among men. Most participants were married or in a marriage-like relationship and had an Argentine nationality.

**Table 1:** Socio-demographic characteristics of participants included in the analysis.

Characteristic	Overall (n = 1,067)	Women (n = 893)	Men (n = 174)
	n (%)	n (%)	n (%)
Age			
18 to 39 years	700 (65.6)	600 (67.2)	100 (57.5)
40 to 49 years	159 (14.9)	129 (14.4)	30 (17.2)
50 to 69 years	208 (19.5)	164 (18.4)	44 (25.3)
Marital status			
Married	352 (33.0)	292 (32.7)	60 (34.5)
Single	543 (50.9)	459 (51.4)	84 (48.3)
Divorced	24 (2.2)	19 (2.1)	5 (2.9)
Separated	109 (10.2)	90 (10.1)	19 (10.9)
Widower	39 (3.7)	33 (3.7)	6 (3.4)
Married or in a marriage-like relationship	744 (69.7)	621 (69.5)	123 (70.7)
Argentine nationality	1,047 (98.1)	875 (98.0)	172 (98.9)

### Prevalence of Mood and Anxiety Disorders

The lifetime, 12-month and 30-day prevalence of any mood or anxiety disorder was 40.4, 20.1, and 7.8%, respectively (**Table 2**). Anxiety disorders were more frequent than mood disorders in the participants’ lifetime and in the last 12 months and 30 days. Social phobia was the most common specific disorder at any time point among participants included in the current analysis, while a severe depressive episode was the most common mood disorder. The lifetime, 12-month and 30-day prevalence of comorbid mood and anxiety disorders was 8.6, 3.5, and 0.4%, respectively

**Table 2:** Lifetime, 12-month and 30-day prevalence of mental disorders among participants included in the current analysis (n = 1,067).

	Lifetime prevalence		12-month prevalence		30-day prevalence	
	n	% (95% CI)	n	% (95% CI)	n	% (95% CI)
Any mood or anxiety disorder	431	40.4 (37.4, 43.4)	215	20.1 (17.8, 22.7)	83	7.8 (6.2, 9.6)
Mood disorders	206	19.3 (17.0, 21.8)	93	8.7 (7.1, 10.6)	25	2.3 (1.5, 3.4)
Mania	37	3.5 (2.4, 4.7)	14	1.3 (0.1, 2.2)	3	0.3 (0.1, 0.8)
Hypomania	43	4.0 (2.9, 5.4)	18	1.7 (1.0, 2.7)	6	0.6 (0.2, 1.2)
Severe depressive episode	72	6.7 (5.3, 8.4)	35	3.3 (2.3, 4.5)	9	0.8 (0.4, 1.6)
Moderate depressive episode	55	5.1 (3.9, 6.7)	28	1.5 (0.1, 2.4)	8	0.8 (0.3, 1.5)
Mild depressive episode	46	4.3 (3.2, 5.7)	16	2.6 (1.8, 3.8)	2	0.2 (0.0, 0.7)
Dysthymia	27	2.5 (1.6, 3.6)	12	1.5 (0.1, 2.4)	3	0.3 (0.1, 0.8)
Anxiety disorders	317	29.7 (27.0, 32.5)	159	14.9 (12.8, 17.2)	62	5.8 (4.5, 7.4)
Agoraphobia without panic disorder	56	5.3 (3.9, 6.8)	27	2.5 (1.7, 3.7)	8	0.8 (0.3, 1.5)
Social phobia	105	9.8 (8.1, 11.8)	54	5.1 (3.8, 6.6)	20	1.9 (1.1, 2.9)
Panic disorder	44	4.1 (3.0, 5.5)	26	2.4 (1.6, 3.5)	11	1.0 (0.5, 1.8)
Generalized anxiety disorder	31	2.9 (1.9, 4.1)	15	1.4 (0.1, 2.3)	4	0.4 (0.1, 1.0)
Obsessive compulsive disorder	23	2.2 (1.4, 3.2)	20	1.9 (1.1, 2.9)	10	0.9 (0.5, 1.7)
Post-traumatic stress disorder	48	4.5 (3.3, 5.9)	13	1.2 (0.7, 2.1)	6	0.6 (0.2, 1.2)
Mood and anxiety disorders (comorbidity)	92	8.6 (7.0, 10.5)	37	3.5 (2.5, 4.7)	4	0.4 (0.1, 1.0)

CI, confidence interval.

There were no statistically significant differences in the lifetime prevalence of mood and anxiety disorders comparing women versus men (**Table 3**, top panel). The prevalence of any mood or anxiety disorder and of any anxiety disorder was higher among women versus men in the last 12 months (**Table 3**, middle panel) and 30 days (**Table 3**, bottom panel). The 12-month and 30-day prevalence of mood disorders and comorbid anxiety and mood disorders was similar among men and women. There were no statistically significant differences in the lifetime, 12-month and 30-day prevalence of mood and anxiety disorders, anxiety disorders, mood disorders, and comorbid anxiety and mood disorders when the analysis was conducted stratified by age groups and being married or in a marriage-like relationship status (**Supplemental Tables 1****–****6**).

**Table 3:** Lifetime, 12-month and 30-day prevalence of mental disorders stratified by sex among participants included in the current analysis (n = 1,067).

	Women (n = 893)		Men (n = 174)		
	n	% (95% CI)	n	% (95% CI)	p-value^a^
***Lifetime prevalence***					
Any mood or anxiety disorder	367	41.1 (37.8, 44.3)	64	36.8 (29.9, 44.3)	0.31
Mood disorders	175	19.6 (17.0, 22.3)	31	17.8 (12.1, 23.6)	0.67
Mania	29	3.2 (2.1, 4.4)	8	4.6 (1.7, 8.0)	0.37
Hypomania	33	3.7 (2.5, 5.0)	10	5.7 (2.3, 9.2)	0.21
Severe depressive episode	64	7.2 (5.6, 9.0)	8	4.6 (1.7, 8.0)	0.25
Moderate depressive episode	49	5.5 (4.0, 7.1)	6	3.4 (1.1, 6.3)	0.35
Mild depressive episode	37	4.1 (2.8, 5.5)	9	5.2 (2.3, 8.6)	0.54
Dysthymia	26	2.9 (1.9, 4.0)	1	0.6 (0.0, 1.7)	0.11
Anxiety disorders	275	30.8 (27.8, 33.9)	42	24.1 (17.8, 30.5)	0.09
Agoraphobia without panic disorder	50	5.6 (4.1, 7.2)	6	3.4 (1.1, 6.3)	0.35
Social phobia	91	10.2 (8.3, 12.2)	14	8.0 (4.0, 12.1)	0.49
Panic disorder	40	4.5 (3.2, 5.9)	4	2.3 (0.6, 4.6)	0.22
Generalized anxiety disorder	29	3.2 (2.1, 4.4)	2	1.1 (0.0, 2.9)	0.21
Obsessive compulsive disorder	19	2.1 (1.2, 3.1)	4	2.3 (0.6, 4.6)	0.78
Post-traumatic stress disorder	44	4.9 (3.6, 6.4)	4	2.3 (0.6, 4.6)	0.16
Mood and anxiety disorders (comorbidity)	83	9.3 (7.5, 11.4)	*9*	5.2 (2.4, 9.6)	0.08
**Twelve-month prevalence**					
Any mood or anxiety disorder	191	21.4 (18.7, 24.1)	24	13.8 (8.6, 19.0)	0.02
Mood disorders	83	9.3 (7.4, 11.2)	10	5.7 (2.3, 9.2)	0.14
Mania	11	1.2 (0.6, 2.0)	3	1.7 (0.0, 4.0)	0.49
Hypomania	14	1.6 (0.8, 2.5)	4	2.3 (0.6, 4.6)	0.52
Severe depressive episode	33	3.7 (2.5, 5.0)	2	1.1 (0.0, 2.9)	0.10
Moderate depressive episode	12	1.3 (0.6, 2.1)	4	2.3 (0.6, 4.6)	0.31
Mild depressive episode	26	2.9 (1.9, 4.0)	2	1.1 (0.0, 2.9)	0.30
Dysthymia	16	1.8 (1.0, 2.7)	0	0.0 (0.0, 2.1)	0.09
Anxiety disorders	142	15.9 (13.5, 18.4)	17	9.8 (5.7, 14.4)	0.04
Agoraphobia without panic disorder	26	2.9 (1.9, 4.0)	1	0.6 (0.0, 1.7)	0.11
Social phobia	47	5.3 (3.9, 6.8)	7	4.0 (1.1, 6.9)	0.58
Panic disorder	23	2.6 (1.6, 3.7)	3	1.7 (0.0, 4.0)	0.79
Generalized anxiety disorder	14	1.6 (0.8, 2.5)	1	0.6 (0.0, 1.7)	0.49
Obsessive compulsive disorder	17	1.9 (1.0, 2.8)	3	1.7 (0.0, 4.0)	> 0.99
Post-traumatic stress disorder	12	1.3 (0.6, 2.1)	1	0.6 (0.0, 1.7)	0.71
Mood and anxiety disorders (comorbidity)	34	3.8 (2.7, 5.3)	3	1.7 (0.0, 4.0)	0.25
**Thirty-day prevalence**					
Any mood or anxiety disorder	76	8.5 (6.8, 10.5)	7	4.0 (1.6, 8.1)	0.04
Mood disorders	22	2.5 (1.6, 3.7)	3	1.7 (0.4, 5.0)	0.78
Mania	3	0.3 (0.1, 0.9)	0	0.0 (0.0, 2.1)	> 0.99
Hypomania	5	0.7 (0.2, 1.5)	1	0.6 (0.0, 3.1)	> 0.99
Severe depressive episode	8	0.9 (0.4, 1.7)	1	0.6 (0.0, 3.1)	> 0.99
Moderate depressive episode	8	0.9 (0.4, 1.7)	0	0.0 (0.0, 2.1)	0.37
Mild depressive episode	1	0.1 (0.0, 0.6)	1	0.6 (0.0, 3.1)	0.30
Dysthymia	3	0.3 (0.1, 0.9)	0	0.0 (0.0, 2.1)	> 0.99
Anxiety disorders	58	6.5 (5.0, 8.3)	4	2.3 (0.6, 5.8)	0.03
Agoraphobia without panic disorder	8	0.9 (0.4, 1.7)	0	0.0 (0.0, 2.1)	0.38
Social phobia	18	2.0 (1.2, 3.2)	2	1.1 (0.1, 4.0)	0.75
Panic disorder	10	1.1 (0.5, 2.0)	1	0.6 (0.0, 3.1)	> 0.99
Generalized anxiety disorder	4	0.4 (0.1, 1.1)	0	0.0 (0.0, 2.1)	> 0.99
Obsessive compulsive disorder	10	1.1 (0.5, 2.0)	0	0.0 (0.0, 2.1)	0.38
Post-traumatic stress disorder	5	0.7 (0.2, 1.5)	1	0.6 (0.0, 3.1)	> 0.99
Mood and anxiety disorders (comorbidity)	4	0.4 (0.1, 1.1)	0	0.0 (0.0, 2.1)	> 0.99

CI, confidence interval.

a Calculated using the Fisher’s exact test.

### Sociodemographic Characteristics Associated With Mood and Anxiety Disorders

**Figure 1:**
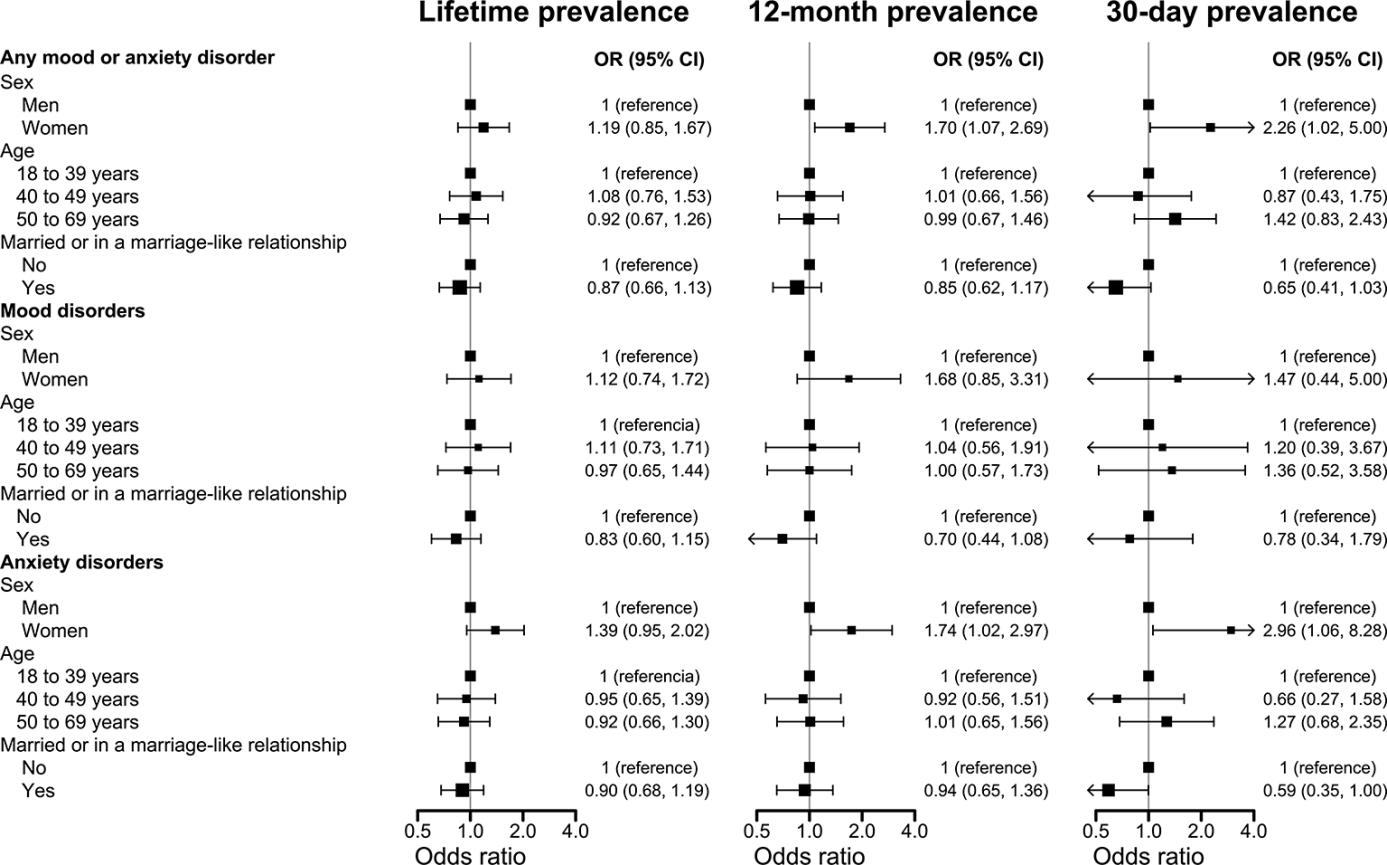
Odds ratios and 95% confidence intervals for mood and anxiety disorders associated with sex, age, and being married or in a marriage-like relationship. CI, confidence interval; OD, odds ratio. Models include adjustment for age, sex and being married or in a marriage-like relationship, simultaneously.

**Figure 1** shows multivariable-adjusted odds ratios for mood and anxiety disorders in the participants’ lifetime and in the last 12 months and 30 days associated with sociodemographic characteristics. Women were more likely to have any mood or anxiety disorder, and any anxiety disorder compared with men, although the difference was numerically lower and not statistically significant when the lifetime prevalence was analyzed. The odds ratio comparing the lifetime, 12-month and 30-days prevalence of mood disorders among women versus men were 1.12 (95% CI 0.74, 1.72), 1.68 (95% CI 0.85, 3.31), and 1.47 (95% CI 0.44, 5.00), respectively. There were no statistically significant differences in the odds of any mood or anxiety disorder, mood disorders or anxiety disorders across groups defined by age or being married or in a marriage-like relationship status.

## Discussion

In the current study, the lifetime, 12-month and 30-day prevalence of any mood or anxiety disorder among adults seeking care for general health problems in public primary healthcare centers in Cordoba city was 40.4% (95% CI 37.4, 43.4%), 20.1% (95% CI 17.8, 22.7%), and 7.8% (95% CI 6.2, 9.6%), respectively. Anxiety disorders were more common than mood disorder in the study population. Also, mood and anxiety disorders were more common among women versus men, particularly in the last 12 months and 30 days.

Goldberg and Lecrubier compared the prevalence of mood and anxiety disorders among people seeking care in primary healthcare services in 15 cities from Latin America, US, Europe, Asia, and Africa using the CIDI, Primary Health Care version (12). In this study, the prevalence of any mood or anxiety disorder varied widely across cities, ranging from 7.3 in Shanghai to 52.5% in Santiago de Chile. The other city from Latin America included in this analysis was Rio de Janeiro, which had the second higher prevalence of mood and anxiety disorders (35.5%) after Santiago de Chile (12). Results from the current study are consistent with the high prevalence of mood and anxiety disorders among people seeking care in primary healthcare services in the two cities from Latin America included in the analysis by Goldberg and Lecrubier and suggest that the prevalence of mood and anxiety disorders may be higher in Latin America as compared with other regions. In the current study, the prevalence of anxiety disorders was higher compared to mood disorders. In the analysis conducted by Goldberg and Lecrubier, the prevalence of anxiety disorders was lower compared to mood disorders in most cities, including Santiago de Chile. However, the prevalence of anxiety disorders was higher compared to mood disorders in Rio de Janeiro.

There are few data available on the prevalence of mental disorders in Argentina. In a cross-sectional analysis of 3,927 adults from the general population conducted in the eight largest metropolitan areas of Argentina, the lifetime and 12-month prevalence of mental disorders as defined using the CIDI was 29.1 and 14.8%, respectively (26, 27). Also, the prevalence of anxiety disorders was higher compared to mood disorders, a finding consistent with the current study. The prevalence of mental disorders in the prior analysis of adults from the general population conducted in Argentina was lower compared with the prevalence of mood and anxiety disorders among adults seeking care for general health problems in public primary healthcare centers in the current study. The higher prevalence of mental disorders in the current study may be explained by individuals with mood and anxiety disorders being more likely to seek medical attention compared with the general population. This is consistent with analyses conducted by Bijl and Ravelli in the Netherlands suggesting that individuals with mental disorders are more likely to seek any form of care, and particularly primary healthcare, compared to individuals without mental disorders (28).

Prior studies suggest that the proportion of adults with mental disorders who do not receive mental treatment (i.e., the treatment gap) is high, including those seeking care in primary healthcare services (26, 29). This is concerning considering that most mental disorders could be effectively treated with current therapies (29). Results from the current study suggest that the prevalence of mood and anxiety disorders in adults seeking care for general health problems in primary healthcare centers in Cordoba city is high. Specifically, about 20.1 and 7.8% of adults seeking care for general health problems in primary healthcare services had a mood or anxiety disorder in the last 12 months and 30 days, respectively. Therefore, integrating mental health services into the primary health care strategy could be an efficient intervention to increase the detection and treatment of individuals with mood and anxiety disorders, contributing to reduce the treatment gap in this population (20, 21).

The lifetime prevalence of mental disorders was not statistically significantly different when compared across age groups in the current study. Goldberg and Lecrubier also reported that the lifetime prevalence of mental disorders did not differ across age subgroups among individuals seeking care in primary healthcare services in their multi-city analysis (12). This finding could be explained by a cohort effect, with younger generations being more likely to have mental disorders. Also, it has been suggested that using self-report may result in a substantial underestimation of the lifetime prevalence of mental disorders due to incomplete recall (30, 31). Older people may be less likely to recall past mental disorder episodes, particularly those occurring in their youth, as compared with younger individuals (31). This differential recall could contribute to explain why the lifetime prevalence of mental disorders did not increase with age in the current analysis. Given the potential limitation of the self-reported lifetime prevalence, the 12-month and 30-day prevalence should be preferred when studying the distribution of mental disorders in epidemiology research.

In the current study, 83.7% of participants were women. The large proportion of women in the current analysis is not explained by a differential participation by sex as only few consultants declined to be included in the study (n=133). Rather, this is consistent with prior studies showing that the majority of consultants in public primary healthcare centers in Argentina are women (32–35). In the current study, women had a higher prevalence of anxiety disorders compared with men. However, the difference was numerically lower and not statistically significant for the lifetime prevalence versus the 12-month and 30-day prevalence. The reasons behind this finding are unclear. A possible explanation for this finding is that women may have longer or more recurrent episodes of anxiety disorders than men. Also, incomplete recall of older episodes may have contributed to attenuate sex differences in the lifetime prevalence of anxiety disorders. The prevalence of mood disorders in the current study was not statistically significantly different between women and men. However, odds ratios for 12-month and 30-day prevalence were well above 1 and 95% CI were very wide, suggesting that the current study may be underpowered to detect a true difference between sex groups. Overall, results from the current study suggest that interventions aimed to integrate mental health services into the primary healthcare strategy may have a greater benefit for women versus men.

The current study included a large number of adults seeking care for general health problems in public primary healthcare centers in Cordoba city. Also, a probabilistic, stratified, multi-stage sampling design was implemented to ensure the representation of neighborhoods with different socioeconomic characteristics. Despite these strengths, the current study has known and potential limitations. Only consultants in primary healthcare centers funded by the local government were included. Therefore, results from the current study may not be generalizable to consultants in primary healthcare centers not funded by the government, including health maintenance organizations. The vast majority of study participants were from Argentina. Therefore, we were not able to analyze the prevalence of mood and anxiety disorders among immigrants, a vulnerable and economically disadvantaged population. No data were collected from the 133 adults who refused to participate of the current study as they did not provide their informed consent, precluding a comparison with those who were included in the analysis. Finally, the study was conducted in Cordoba city, and may not be generalizable to other cities in Argentina.

## Conclusion

In the current study, the prevalence of mood and anxiety disorders was high among adults seeking care for general health problems in public primary healthcare centers in Cordoba city. The prevalence of mood and anxiety disorders found in the current study was higher compared with the prevalence of mental disorders among adults from the general population in large metropolitan areas of Argentina. Results from the current study suggest that integrating mental health services into the primary health care strategy could contribute to reduce the treatment gap and improve the quality of care among individuals with mood and anxiety disorders, particularly in women.

## Data Availability Statement

The datasets generated for this study are available upon justified request to the corresponding author.

## Ethics Statement

The studies involving human participants were reviewed and approved by the Hospital Nacional de Clínicas Institutional Review Board, Córdoba, Argentina. The patients/participants provided their written informed consent to participate in this study.

## Author Contributions

MB contributed to the study concept and design, obtention of funds, acquisition, analysis and interpretation of data, and drafting of the manuscript. RA, JE, and RF contributed to the study concept and design, interpretation of data, and critical revision of the manuscript for important intellectual content. LC, RAA, EV, and ES contributed to the interpretation of data and critical revision of the manuscript for important intellectual content. All co-authors have contributed to the submitted manuscript and met the International Committee of Medical Journal Editors (ICMJE) criteria for authorship. All authors have read and approved the final version of the present manuscript.

## Funding

The present study was funded by research grants from the Facultad de Ciencias Médicas de la Universidad Nacional de Córdoba, Argentina (PROMED), and the Florencio Fiorini Foundation, Argentine Academy of Medicine. This study was endorsed by Secretaría de Ciencia y Técnica (SeCyT), Universidad Nacional de Córdoba, Argentina. We have no other disclosures to report.

## Conflict of Interest

The authors declare that the research was conducted in the absence of any commercial or financial relationships that could be construed as a potential conflict of interest.
