# Supplementary material for: Prevalence of Mood and Anxiety Disorders Among Adults Seeking Care in Primary Healthcare Centers in Cordoba, Argentina
Source: Front Psychiatry. 2020 Mar 27;11:232. doi: 10.3389/fpsyt.2020.00232 (PMC7118212; doi:10.3389/fpsyt.2020.00232)
Supplement: Supplementary file 1 [file DataSheet_1.docx]

**Prevalence of mood and anxiety disorders among adults seeking care in primary healthcare centers in Cordoba, Argentina.**

**SUPPLEMENTAL MATERIAL**

María Soledad Burrone^1,2*^, Rubén Alvarado^1,3^, Lisandro D. Colantonio^4^, Julio E. Enders^2^, Roberto Ariel Abeldaño Zuñiga^5^, Eliecer Valencia^1,6^, Ezra Susser^6,7^, Ruth A. Fernández^2^

1. Instituto de Ciencias de la Salud, Universidad de O´Higgins, Rancagua, Chile.
2. Escuela de Salud Pública, Facultad de Ciencias Médicas de la Universidad Nacional de Córdoba, Córdoba, Argentina.
3. Unit of Mental Health, School of Public Health, Faculty of Medicine, University of Chile, Santiago, Chile.
4. Department of Epidemiology, School of Public Health, University of Alabama at Birmingham, Birmingham, AL, USA.
5. División de Estudios de Posgrado, Universidad de la Sierra Sur – Consejo Nacional de Ciencia y Tecnología. Oaxaca, México
6. Mailman School of Public Health, Department of Epidemiology, Columbia University, New York, NY, USA.
7. Division of Health Policy and Behavioural Science, New York State Psychiatric Institute, New York, NY, USA.

*** Correspondence**:

Burrone, María Soledad.

Address: Av. Libertador Bernardo O'Higgins 609 (ex 611), Rancagua, Chile. Telephone: (+56) 229030000, Zip code: 2820000.

Email: [mariasoledad.burrone@uoh.cl](mailto:mariasoledad.burrone@uoh.cl)

**Supplemental Table 1.** Lifetime prevalence of mood and anxiety disorders stratified by age among participants included in the current analysis (n=1,067).

|  | | **18-39 years (n= 700)** | | **40-49 years (n=159)** | | **50-69 years (n=208)** | | **p-value^a^** |
| --- | --- | --- | --- | --- | --- | --- | --- | --- |
|  | | **n** | **% (95% CI)** | **n** | **% (95% CI)** | **n** | **% (95% CI)** |  |
| ***Any mood or anxiety disorder*** | | ***284*** | ***40.6 (36.9, 44.3)*** | ***67*** | ***42.1 (34.4, 50.2)*** | ***80*** | ***38.5 (31.8, 45.4)*** | ***0.77*** |
| ***Mood disorders*** | | ***134*** | ***19.1 (16.3, 22.2)*** | ***33*** | ***20.8 (14.7, 27.9)*** | ***39*** | ***18.8 (13.7, 24.7)*** | ***0.88*** |
|  | Mania | 27 | 3.9 (2.6, 5.6) | 4 | 2.5 (0.7, 6.3) | 6 | 2.9 (1.1, 6.2) | 0.72 |
|  | Hypomania | 29 | 4.1 (2.8, 5.9) | 6 | 3.8 (1.4, 8.0) | 8 | 3.8 (1.7, 7.4) | >0.99 |
|  | Severe depressive episode | 49 | 7.0 (5.2, 9.1) | 11 | 6.9 (3.5, 12.0) | 12 | 5.8 (3.0, 9.9) | 0.84 |
|  | Moderate depressive episode | 33 | 4.7 (3.3, 6.6) | 5 | 3.1 (1.0, 7.2) | 17 | 8.2 (4.8, 12.8) | 0.08 |
|  | Mild depressive episode | 31 | 4.4 (3.0, 6.2) | 11 | 6.9 (3.5, 12.0) | 4 | 1.9 (0.1, 4.9) | 0.06 |
|  | Dysthymia | 20 | 2.9 (1.8, 4.4) | 1 | 0.6 (0.0, 3.4) | 6 | 2.9 (1.1, 6.2) | 0.25 |
| ***Anxiety disorders*** | | ***212*** | ***30.3 (26.9, 33.8)*** | ***46*** | ***28.9 (22.0, 36.6)*** | ***59*** | ***28.4 (22.3, 35.0)*** | ***0.86*** |
|  | Agoraphobia without panic disorder | 39 | 5.6 (4.0, 7.5) | 9 | 5.7 (2.6, 10.5) | 8 | 3.8 (1.7, 7.4) | 0.62 |
|  | Social phobia | 70 | 10.0 (7.9, 12.5) | 15 | 9.4 (5.4, 15.1) | 20 | 9.6 (6.0, 14.5) | >0.99 |
|  | Panic disorder | 33 | 4.7 (3.3, 6.6) | 6 | 3.8 (1.4, 8.0) | 5 | 2.4 (0.7, 5.5) | 0.36 |
|  | Generalized anxiety disorder | 31 | 3.0 (1.9, 4.5) | 5 | 3.1 (1.0, 7.2) | 5 | 2.4 (0.7, 5.5) | 0.92 |
|  | Obsessive compulsive disorder | 15 | 2.1 (1.2, 3.5) | 1 | 0.6 (0.0, 3.5) | 7 | 3.4 (1.4, 6.8) | 0.21 |
|  | Post-traumatic stress disorder | 32 | 4.6 (3.1, 6.4) | 5 | 3.1 (1.0, 7.2) | 11 | 5.3 (2.7, 9.3) | 0.64 |
| ***Mood and anxiety disorders (comorbidity)*** | | ***62*** | ***8.9 (6.9, 11.2)*** | ***12*** | ***7.5 (4.0, 12.8)*** | ***18*** | ***8.7 (5.2, 13.3)*** | ***0.92*** |

CI: confidence interval.

^a^ Calculated using the Fisher’s exact test.

**Supplemental Table 2:** Twelve-month prevalence of mood and anxiety disorders stratified by age among participants included in the current analysis (n=1,067).

|  | | **18-39 years (n= 700)** | | **40-49 years (n=159)** | | **50-69 years (n=208)** | | **p-value^a^** |
| --- | --- | --- | --- | --- | --- | --- | --- | --- |
|  | | **n** | **% (95% CI)** | **n** | **% (95% CI)** | **n** | **% (95% CI)** |  |
| ***Any mood or anxiety disorder*** | | ***142*** | ***20.3 (17.4, 23.5)*** | ***32*** | ***20.1 (14.2, 27.2)*** | ***41*** | ***19.7 (14.5, 25.8)*** | ***0.99*** |
| ***Mood disorders*** | | ***61*** | ***8.7 (6.7, 11.1)*** | ***14*** | ***8.8 (4.9, 14.3)*** | ***18*** | ***8.7 (5.2, 13.3)*** | ***>0.99*** |
|  | Mania | 11 | 1.6 (0.8, 2.8) | 2 | 1.3 (0.2, 4.5) | 1 | 0.5 (0.0, 2.6) | 0.52 |
|  | Hypomania | 13 | 1.9 (1.0, 3.2) | 3 | 1.9 (0.4, 5.4) | 2 | 1.0 (0.1, 3.4) | 0.77 |
|  | Severe depressive episode | 28 | 4.0 (2.7, 5.7) | 4 | 2.5 (0.7, 6.3) | 3 | 1.4 (0.2, 4.2) | 0.17 |
|  | Moderate depressive episode | 16 | 2.3 (1.3, 3.7) | 2 | 1.3 (0.2, 4.5) | 10 | 4.8 (2.3, 8.7) | 0.09 |
|  | Mild depressive episode | 9 | 1.3 (0.6, 2.4) | 5 | 3.1 (0.1, 7.2) | 2 | 1.0 (0.1, 3.4) | 0.21 |
|  | Dysthymia | 12 | 1.7 (0.8, 3.0) | 0 | 0.0 (0.0, 0.2) | 4 | 1.9 (0.5, 4.9) | 0.23 |
| ***Anxiety disorders*** | | ***106*** | ***15.1 (12.6, 18.0)*** | ***22*** | ***13.8 (8.9, 20.2)*** | ***31*** | ***14.9 (10.3, 20.5)*** | ***0.95*** |
|  | Agoraphobia without panic disorder | 18 | 2.6 (1.5, 4.0) | 3 | 1.9 (0.4, 5.4) | 6 | 2.9 (1.1, 6.2) | 0.84 |
|  | Social phobia | 35 | 5.0 (2.2, 9.7) | 8 | 5.0 (2.2, 9.7) | 11 | 5.3 (2.7, 9.2) | 0.98 |
|  | Panic disorder | 20 | 2.9 (1.8, 4.4) | 4 | 2.5 (0.7, 6.3) | 2 | 1.0 (0.1, 3.4) | 0.32 |
|  | Generalized anxiety disorder | 8 | 1.1 (0.4, 2.2) | 3 | 1.9 (0.4, 5.4) | 4 | 1.9 (0.5, 4.9) | 0.50 |
|  | Obsessive compulsive disorder | 13 | 1.9 (1.0, 3.2) | 1 | 0.6 (0.0, 3.5) | 6 | 2.9 (1.1, 6.2) | 0.30 |
|  | Post-traumatic stress disorder | 7 | 1.0 (0.4, 2.0) | 2 | 1.3 (0.2, 4.5) | 4 | 1.9 (0.5, 4.9) | 0.45 |
| ***Mood and anxiety disorders (comorbidity)*** | | ***25*** | ***3.6 (2.3, 5.2)*** | ***4*** | ***2.5 (0.7, 6.3)*** | ***8*** | ***3.8 (1.7, 7.4)*** | ***0.80*** |

CI: confidence interval.

^a^ Calculated using the Fisher’s exact test.

**Supplemental Table 3:** Thirty-day prevalence of mood and anxiety disorders stratified by age among participants included in the current analysis (n=1,067).

|  | | **18-39 years (n= 700)** | | **40-49 years (n=159)** | | **50-69 years (n=208)** | | **p-value^a^** |
| --- | --- | --- | --- | --- | --- | --- | --- | --- |
|  | | **n** | **% (95% CI)** | **n** | **% (95% CI)** | **n** | **% (95% CI)** |  |
| ***Any mood or anxiety disorder*** | | ***52*** | ***7.4 (5.6, 9.6)*** | ***10*** | ***6.3 (3.0, 11.3)*** | ***21*** | ***10.1 (6.4, 15.0)*** | ***0.35*** |
| ***Mood disorders*** | | ***15*** | ***2.1 (1.2, 3.5)*** | ***4*** | ***2.5 (0.6, 6.2)*** | ***6*** | ***2.9 (1.1, 6.2)*** | ***0.79*** |
|  | Mania | 2 | 0.3 (0.0, 1.0) | 0 | 0.0 (0.0, 2.3) | 1 | 0.5 (0.0, 2.6) | 0.72 |
|  | Hypomania | 4 | 0.6 (0.2, 1.5) | 0 | 0.0 (0.0, 2.3) | 2 | 1.0 (0.1, 3.4) | 0.58 |
|  | Severe depressive episode | 5 | 0.7 (0.2, 1.7) | 2 | 1.3 (0.2, 4.5) | 2 | 1.0 (0.1, 3.4) | 0.69 |
|  | Moderate depressive episode | 6 | 0.9 (0.3, 1.9) | 0 | 0.0 (0.0, 2.3) | 2 | 1.0 (0.1, 3.4) | 0.66 |
|  | Mild depressive episode | 0 | 0.0 (0.0, 0.5) | 2 | 1.3 (0.2, 4.5) | 0 | 0.0 (0.0, 1.8) | 0.02 |
|  | Dysthymia | 2 | 0.3 (0.0, 1.0) | 0 | 0.0 (0.0, 2.3) | 1 | 0.5 (0.0, 2.6) | 0.71 |
| ***Anxiety disorders*** | | ***41*** | ***5.8 (4.2, 7.9)*** | ***6*** | ***3.8 (1.4, 8.0)*** | ***15*** | ***7.2 (4.1, 11.6)*** | ***0.39*** |
|  | Agoraphobia without panic disorder | 6 | 0.9 (0.3, 1.9) | 1 | 0.6 (0.0, 3.5) | 1 | 0.5 (0.0, 2.6) | >0.99 |
|  | Social phobia | 15 | 2.1 (1.2, 3.5) | 1 | 0.6 (0.0, 3.5) | 4 | 1.9 (0.5, 4.9) | 0.56 |
|  | Panic disorder | 10 | 1.4 (0.7, 2.6) | 1 | 0.6 (0.0, 3.5) | 0 | 0.0 (0.0, 1.8) | 0.17 |
|  | Generalized anxiety disorder | 2 | 0.3 (0.0, 1.0) | 0 | 0.0 (0.0, 2.3) | 2 | 1.0 (0.1, 3.4) | 0.27 |
|  | Obsessive compulsive disorder | 6 | 0.9 (0.3, 1.9) | 0 | 0.0 (0.0, 2.3) | 4 | 1.9 (0.5, 4.9) | 0.18 |
|  | Post-traumatic stress disorder | 4 | 0.6 (0.2, 1.5) | 1 | 0.6 (0.0, 3.5) | 1 | 0.5 (0.0, 2.6) | >0.99 |
| ***Mood and anxiety disorders (comorbidity)*** | | ***4*** | ***0.6 (0.2, 1.5)*** | ***0*** | ***0.0 (0.0, 2.3)*** | ***0*** | ***0.0 (0.0, 1.8)*** | ***0.78*** |

CI: confidence interval.

^a^ Calculated using the Fisher’s exact test.

**Supplemental Table 4:** Lifetime prevalence of mood and anxiety disorders stratified by marital status among participants included in the current analysis (n=1,067).

|  | | **Married or in a marriage-like relationship** | | | |  |
| --- | --- | --- | --- | --- | --- | --- |
|  | | **Yes (n= 744)** | | **No (n=323)** | |  |
|  | | **n** | **% (95% CI)** | **n** | **% (95% CI)** | **p-value^a^** |
| ***Any mood or anxiety disorder*** | | ***293*** | ***39.4 (35.9, 43.0)*** | ***138*** | ***42.7 (37.3, 48.3)*** | ***0.31*** |
| ***Mood disorders*** | | ***137*** | ***18.4 (15.7, 21.4)*** | ***69*** | ***21.4 (17.0, 26.2)*** | ***0.27*** |
|  | Mania | 25 | 3.4 (2.2, 4.9) | 12 | 3.7 (1.9, 6.4) | 0.86 |
|  | Hypomania | 29 | 3.9 (2.6, 5.6) | 14 | 4.3 (2.4, 7.2) | 0.74 |
|  | Severe depressive episode | 48 | 6.5 (4.8, 8.4) | 24 | 7.2 (4.8, 10.9) | 0.60 |
|  | Moderate depressive episode | 35 | 4.7 (3.3, 6.5) | 20 | 6.2 (3.8, 9.4) | 0.37 |
|  | Mild depressive episode | 31 | 4.2 (2.8, 5.9) | 15 | 4.6 (2.6, 7.5) | 0.74 |
|  | Dysthymia | 17 | 2.3 (1.3, 3.6) | 10 | 3.1 (1.5, 5.6) | 0.52 |
| ***Anxiety disorders*** | | ***216*** | ***29.0 (25.8, 32.4)*** | ***101*** | ***31.3 (26.3, 36.6)*** | ***0.47*** |
|  | Agoraphobia without panic disorder | 38 | 5.1 (3.6, 6.9) | 18 | 5.6 (3.3, 8.7) | 0.77 |
|  | Social phobia | 73 | 9.8 (7.8, 12.2) | 32 | 9.9 (6.9, 13.7) | >0.99 |
|  | Panic disorder | 31 | 4.2 (2.8, 5.9) | 13 | 4.0 (2.2, 6.8) | >0.99 |
|  | Generalized anxiety disorder | 17 | 2.3 (1.3, 3.6) | 14 | 4.3 (2.4, 7.2) | 0.08 |
|  | Obsessive compulsive disorder | 16 | 2.2 (1.2, 3.5) | 7 | 2.2 (0.1, 4.4) | >0.99 |
|  | Post-traumatic stress disorder | 28 | 3.8 (2.5, 5.4) | 20 | 6.2 (3.8, 9.4) | 0.11 |
| ***Mood and anxiety disorders (comorbidity)*** | | ***60*** | ***8.1 (6.2, 10.3)*** | ***32*** | ***9.9 (6.9, 13.7)*** | ***0.34*** |

CI: confidence interval.

^a^ Calculated using the Fisher’s exact test.

**Supplemental Table 5:** Twelve-month prevalence of mood and anxiety disorders stratified by marital status among participants included in the current analysis (n=1,067).

|  | | **Married or in a marriage-like relationship** | | | |  |
| --- | --- | --- | --- | --- | --- | --- |
|  | | **Yes (n= 744)** | | **No (n=323)** | | **p-value^a^** |
|  | | **n** | **% (95% CI)** | **n** | **% (95% CI)** |  |
| ***Any mood or anxiety disorder*** | | ***144*** | ***19.5 (16.6, 22.4)*** | ***71*** | ***22.0 (17.6, 26.9)*** | ***0.36*** |
| ***Mood disorders*** | | ***58*** | ***7.8 (6.0, 10.0)*** | ***35*** | ***10.8 (7.8, 14.7)*** | ***0.12*** |
|  | Mania | 8 | 1.1 (0.4, 2.1) | 6 | 1.9 (0.7, 3.9) | 0.40 |
|  | Hypomania | 11 | 1.5 (0.7, 2.6) | 7 | 2.2 (0.9, 4.4) | 0.44 |
|  | Severe depressive episode | 19 | 2.6 (1.5, 4.0) | 16 | 5.0 (2.9, 7.9) | 0.06 |
|  | Moderate depressive episode | 18 | 2.4 (1.4, 3.8) | 10 | 3.1 (1.5, 5.6) | 0.54 |
|  | Mild depressive episode | 12 | 1.6 (0.8, 2.8) | 4 | 1.2 (0.3, 3.1) | 0.78 |
|  | Dysthymia | 10 | 1.3 (0.6, 2.4) | 6 | 1.9 (0.7, 4.0) | 0.59 |
| ***Anxiety disorders*** | | ***109*** | ***14.7 (12.2, 17.4)*** | ***50*** | ***15.5 (11.7, 19.9)*** | ***0.71*** |
|  | Agoraphobia without panic disorder | 16 | 2.2 (1.2, 3.5) | 11 | 3.4 (1.7, 6.0) | 0.29 |
|  | Social phobia | 37 | 5.0 (3.5, 6.8) | 17 | 5.3 (3.1, 8.3) | 0.88 |
|  | Panic disorder | 18 | 2.4 (1.4, 3.8) | 8 | 2.5 (1.1, 4.8) | >0.99 |
|  | Generalized anxiety disorder | 9 | 1.2 (0.6, 2.3) | 6 | 1.9 (0.7, 4.0) | 0.41 |
|  | Obsessive compulsive disorder | 14 | 1.9 (0.1, 3.1) | 6 | 1.9 (0.7, 4.0) | >0.99 |
|  | Post-traumatic stress disorder | 7 | 0.9 (0.4, 1.9) | 6 | 1.9 (0.7, 4.0) | 0.23 |
| ***Mood and anxiety disorders (comorbidity)*** | | ***23*** | ***3.1 (2.0, 4.6)*** | ***14*** | ***4.3 (2.4, 7.2)*** | ***0.36*** |

CI: confidence interval.

^a^ Calculated using the Fisher’s exact test.

**Supplemental Table 6:** Thirty-day prevalence of mood and anxiety disorders stratified by marital status among participants included in the current analysis (n=1,067).

|  | | **Married or in a marriage-like relationship** | | | |  |
| --- | --- | --- | --- | --- | --- | --- |
|  | | **Yes (n= 744)** | | **No (n=323)** | | **p-value^a^** |
|  | | **n** | **% (95% CI)** | **n** | **% (95% CI)** |  |
| ***Any mood or anxiety disorder*** | | ***50*** | ***6.7 (5.0, 8.8)*** | ***33*** | ***10.2 (0.7, 14.0)*** | ***0.06*** |
| ***Mood disorders*** | | ***16*** | ***2.2 (1.2, 3.5)*** | ***9*** | ***2.8 (1.3, 5.2)*** | ***0.52*** |
|  | Mania | 2 | 0.3 (0.0, 1.0) | 1 | 0.3 (0.0, 1.7) | >0.99 |
|  | Hypomania | 4 | 0.5 (0.1, 1.4) | 2 | 0.6 (0.1, 2.2) | >0.99 |
|  | Severe depressive episode | 5 | 0.7 (0.2, 1.6) | 4 | 1.2 (0.3, 3.1) | 0.47 |
|  | Moderate depressive episode | 6 | 0.8 (0.2, 1.7) | 2 | 0.6 (0.1, 2.2) | >0.99 |
|  | Mild depressive episode | 1 | 0.1 (0.0, 0.7) | 1 | 0.3 (0.0, 1.7) | 0.51 |
|  | Dysthymia | 3 | 0.4 (0.0, 1.2) | 0 | 0.0 (0.0, 1.1) | 0.56 |
| ***Anxiety disorders*** | | ***36*** | ***4.8 (3.4, 6.6)*** | ***26*** | ***8.0 (5.3, 11.6)*** | ***0.05*** |
|  | Agoraphobia without panic disorder | 5 | 0.7 (0.2, 1.6) | 3 | 0.9 (1.9, 2.7) | 0.70 |
|  | Social phobia | 10 | 1.3 (0.6, 2.5) | 10 | 3.1 (1.5, 5.6) | 0.08 |
|  | Panic disorder | 5 | 0.7 (0.2, 1.6) | 6 | 1.9 (0.7, 4.0) | 0.09 |
|  | Generalized anxiety disorder | 1 | 0.1 (0.0, 0.7) | 3 | 0.9 (0.2, 2.7) | 0.09 |
|  | Obsessive compulsive disorder | 7 | 0.9 (0.2, 2.7) | 3 | 0.9 (0.2, 2.7) | >0.99 |
|  | Post-traumatic stress disorder | 2 | 0.3 (0.0, 1.0) | 4 | 1.2 (0.3, 3.1) | 0.07 |
| ***Mood and anxiety disorders (comorbidity)*** | | ***2*** | ***0.3 (0.0, 1.0)*** | ***2*** | ***0.6 (0.1, 2.2)*** | ***0.59*** |

CI: confidence interval.

^a^ Calculated using the Fisher’s exact test.
